# Multimodality imaging of anomalous pulmonary veins

**DOI:** 10.1186/1476-7120-9-3

**Published:** 2011-02-02

**Authors:** Roman Nepomuceno, Matthew Zeglinski, Jordyn Lerner, Wlodzimierz Czarnecki, Iain DC Kirkpatrick, Jacek Strzelczyk, Davinder S Jassal

**Affiliations:** 1 Institute of Cardiovascular Sciences, St. Boniface Research Centre, University of Manitoba, Winnipeg, Manitoba, Canada; 2 Section of Cardiology, Department of Internal Medicine, University of Manitoba, Winnipeg, Manitoba, Canada; 3 Department of Radiology, University of Manitoba, Winnipeg, Manitoba, Canada

## Abstract

Partial anomalous pulmonary venous connection (PAPVC) is an extremely rare congenital condition where one or more of the pulmonary veins are connected to the venous circulation. Although initially suspected with unexplained right ventricular enlargement on transthoracic echocardiography (TTE), cardiac MRI is able to delineate the anatomical variant. We present a case of a 65-year-old male diagnosed with left sided PAPVC using multimodality cardiac imaging.

## Case Report

**Figure 1:**
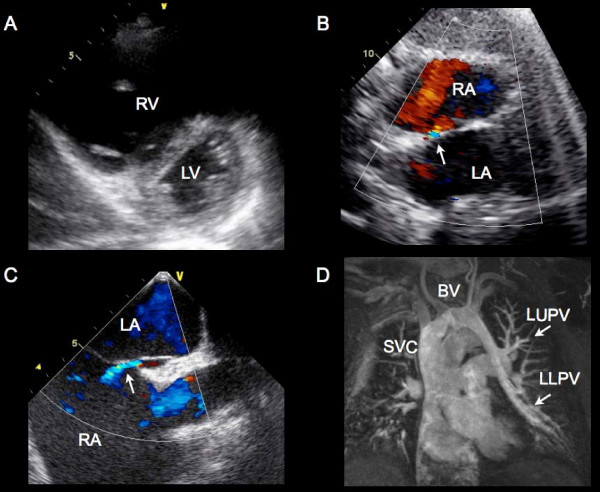
**Multimodality imaging of anomalous pulmonary veins**: **A) **A parasternal short axis view on TTE demonstrating moderate RV enlargement. RV, right ventricle; LV, left ventricle. **B) **A subxiphoid view on TTE demonstrating left to right shunting across the interatrial septum on color Doppler, consistent with a PFO. RA, right atrium; LA, left atrium. **C) **A midesophageal view on TEE demonstrating left to right shunting across the interatrial septum on color Doppler, consistent with a PFO. RA, right atrium; LA, left atrium. **D) **Cardiac magnetic resonance imaging (CMR) demonstrated a congenital partial anomalous connection of both left pulmonary veins to the innominate vein. SVC, superior vena cava; BV, brachiocephalic vein; LUPV, left upper pulmonary vein; LLPV, left lower pulmonary vein.

A 65-year-old male with diabetes, hyperlipidemia, and hypertension presented with palpitations in the absence of dyspnea, chest pain, nor syncope. On physical examination, the patient was hypertensive with a blood pressure of 167/125 mmHg. The jugular venous pressure (JVP) and carotid contour were normal. There was evidence of a parasternal lift, consistent with right ventricular (RV) enlargement with no evidence of cardiac murmurs. Transthoracic echocardiography (TTE) confirmed moderate RV enlargement with preserved systolic function. A small patent foramen ovale (PFO) was identified with left-to-right shunting on color Doppler (Figure 1A and 1B, Additional File 1, Movie 1A and Additional File 2, Movie 1B). Transesophageal echocardiography (TEE) confirmed the left-to-right shunt across the PFO by color Doppler with intermittent right-to-left flow following administration of agitated saline contrast (Figure 1C, Additional File 3, Movie 1C). However, the degree of RV enlargement on both TTE and TEE was disproportionate to the interatrial shunt. Cardiac magnetic resonance imaging (CMR) demonstrated a congenital partial anomalous connection of both left pulmonary veins to the innominate vein as the etiology of RV enlargement (Figure 1D).

## Discussion

Partial anomalous pulmonary venous connection (PAPVC) is an extremely rare congenital condition where one or more of the pulmonary veins are connected to the venous circulation. Its prevalence within the general population is 0.4-0.7% [1]. Approximately 90% of all PAPVC's originate from the right lung, 7% originate from the left lung, and 3% of patients are found to have bilateral PAPVC's originating from both lungs connecting to either the superior vena cava (SVC), the inferior vena cava (IVC), the right atrium or the innominate vein [2]. Common congenital cardiac conditions associated with PAPVC include sinus venosum and ASD secundum defects [1-4]. Clinical presentation of PAPVC may include dyspnea, chest pain, fatigue, syncope, and/or a decreased exercise tolerance [1-4]. On physical examination, individuals with PAPVC may present with an elevated JVP, parasternal lift due to RV enlargement, a right sided S3 and pulmonary hypertension [1-4].

Echocardiography is the initial modality of choice for the noninvasive detection of PAPVC [1,5]. Transthoracic echocardiography (TTE) identifies right atrial and RV enlargement, flattening of the interventricular septum in systole and diastole due to RV pressure overload, elevated pulmonary pressures [6], and/or the presence of an interatrial shunt on color Doppler. In addition to PAPVC, other cardiac entities to consider in the differential diagnosis of unexplained RV enlargement on echocardiography include sinus venosus defect, ostium primum or ASD secundum, or arrythmogenic right ventricular dysplasia. Although TTE is unable to characterize the anatomy of the pulmonary veins, TEE is able to confirm the presence or absence of a PAPVC [1]. If all four pulmonary veins are not identified emptying into the LA on TEE [1], CMR may provide complementary information [7,8]. Due to the higher spatial resolution and increased field of view of CMR, the presence or absence of a PAPVC and quantification of a shunt can be easily identified [7,8].

Individuals with a Qp/Qs shunt ratio higher than 1.5:1 with RV enlargement should undergo surgical correction of the PAPVC [9]. A pulmonary vein can be connected through a baffle or directly back to the left atrium, with surgical correction of a concomitant ASD with a patch if required [9]. In patients with PAPVC, the right atrium may require a larger prosthetic or pericardial patch for repair than an isolated ASD repair, which may cause complications including sick sinus syndrome, variable AV block or obstruction of the superior vena cava [9]. In our case, the patient was asymptomatic with a Qp/Qs of 1.1:1 on CMR, and hence followed conservatively on an annual basis.

## Conclusion

In a patient with unexplained RV enlargement disproportionate to the presence of a small PFO, PAPVC should be considered in the differential diagnosis. Multimodality cardiac imaging using echocardiography and CMR may provide a comprehensive noninvasive evaluation of PAPVC.

## Consent

Written informed consent was obtained from the patient for publication of this case report and accompanying images. A copy of the written consent is available for review by the Editor-in-Chief of this journal.

## Competing interests

The authors declare that they have no competing interests.

## Authors' contributions

RN, MZ, JL, WC, JS, IK, and DJ contributed to the writing of the manuscript. All authors read and approved the final manuscript.

## Supplementary Material

Additional file 1 **A parasternal short axis view on TTE demonstrating RV enlargement**. Movie 1A. Click here for file

Additional file 2 **A subxiphoid view on TTE demonstrating a PFO with left to right shunting on color Doppler**. Movie 1B. Click here for file

Additional file 3 **A midesophageal view on TEE demonstrating a PFO with left to right shunting on color Doppler**. Movie 1C. Click here for file
